# Heterogeneity in the association between gut microbiota and insomnia moderated by Parkinson’s disease status

**DOI:** 10.3389/fcimb.2026.1691665

**Published:** 2026-05-05

**Authors:** Sheng-Hsuan Lin, Ru-Jen Lin, Ruey-Yi Chang, Yu-Fang Huang, Yi-Yung Hsu, Chia-Ling Chu, Yan-Lin Chen, Chong-You Lin, Shih-Chen Fu

**Affiliations:** 1 Institute of Statistics, National Yang Ming Chiao Tung University, Hsinchu, Taiwan; 2 Institute of Data Science and Engineering, National Yang Ming Chiao Tung University, Hsinchu, Taiwan; 3 Department of Biochemistry and Molecular Medicine, National Dong Hwa University, Hualien, Taiwan; 4 Department of Applied Mathematics, National Dong Hwa University, Hualien, Taiwan; 5 Department of Neurology, National Taiwan University Hospital Hsin-Chu Branch, Hsinchu, Taiwan; 6 Department of Biomedical Engineering and Environmental Sciences, National Tsing Hua University, Hsinchu, Taiwan

**Keywords:** alpha and beta diversity, gut microbiome, gut-brain axis, insomnia, Parkinson’s disease

## Abstract

**Introduction:**

Parkinson’s disease (PD) is frequently accompanied by insomnia, and emerging evidence suggests the gut microbiome may play a role. This study investigates gut microbiome differences associated with insomnia in PD patients compared to non-PD individuals.

**Methods:**

We analyzed 310 participants (185 PD patients, 125 controls) categorized by insomnia status. Gut microbiome profiles were obtained using 16S rRNA sequencing and processed with DADA2 using the SILVA database for taxonomic assignment. Alpha and beta diversity analyses and differential abundance analysis were conducted, and functional prediction was performed using PICRUSt2, adjusting for relevant confounders.

**Results:**

Insomnia was linked to higher alpha diversity in non-PD individuals but lower alpha diversity in PD patients. Interaction analysis confirmed distinct associations between insomnia and microbial diversity in the two groups. Differential abundance analysis identified unique insomnia-associated bacterial genera, with differing insomnia-risk-reducing and insomnia-risk-increasing taxa. Functional analysis showed six enriched pathways in controls but only two in PD patients, with no overlap.

**Discussion:**

These findings suggest that insomnia in PD is associated with distinct gut microbiome profiles compared with non-PD individuals. The results highlight the importance of considering disease context when examining microbiome–sleep relationships and may inform future research on microbiome-based approaches for sleep disturbances in PD.

## Introduction

Parkinson’s disease (PD) is a prevalent neurodegenerative disorder with a rapidly increasing global burden. From 1990 to 2015, the age-standardized rates of PD-related deaths, prevalence, and disability-adjusted life years (DALYs) rose significantly, with the number of PD cases globally increasing by 118% to 6.2 million (Feigin et al., 2017; Jiang et al., 2022). This growing prevalence places immense economic and healthcare demands on society, not only through direct medical expenses but also by significantly reducing the quality of life for patients and their families (Kowal et al., 2013). In addition to its motor symptoms, PD is characterized by a wide range of non-motor symptoms that further contribute to patient burden. Among the non-motor symptoms of PD, insomnia is particularly prevalent, affecting an estimated 55.7% of patients—a prevalence three to four times higher than that in the general population (Lebrun et al., 2019). Insomnia in PD not only profoundly impairs quality of life and daily functioning but is also recognized as a critical risk factor for exacerbating PD-related symptoms (Bohnen and Hu, 2019; Duan et al., 2025). Understanding the mechanisms underlying insomnia in PD is essential for optimizing treatment strategies, mitigating the disease burden, and improving patient outcomes.

Recent research has increasingly emphasized the bidirectional relationship between neurodegenerative diseases and sleep disturbances. For example, studies in Alzheimer’s disease (AD) demonstrate that disrupted sleep patterns exacerbate neurodegenerative processes (Ju et al., 2014), while epidemiological evidence suggests that insomnia significantly increases the risk of developing neurodegenerative disorders (Yaffe et al., 2014). In PD, treatments such as dopaminergic agents or amantadine have dual effects on sleep: they can improve nighttime motor symptoms and enhance sleep quality, yet higher doses may worsen insomnia symptoms (Barone et al., 2009). Given this limitation, there is a pressing need to explore alternative therapeutic approaches for managing sleep disturbances in PD patients. Emerging evidence points to the gut microbiome as a critical regulator of sleep and mental states via the microbiome-gut-brain axis. Gut microbiota-derived neurotoxic metabolites, such as D-lactic acid and ammonia, are known to affect brain function and sleep architecture through bidirectional communication along this axis (Bonaz et al., 2013). Despite increasing interest in the gut microbiome’s role in sleep regulation, little is known about its relationship with insomnia in PD patients. Moreover, it remains unclear whether this relationship differs between PD patients and healthy individuals. These knowledge gaps hinder a comprehensive understanding of the mechanisms underlying insomnia in PD and limit progress toward developing targeted therapies.

This study aims to explore the relationship between insomnia and the gut microbiome in PD patients and to compare this relationship with that observed in healthy individuals. Specifically, we seek to (1) analyze the association between gut microbial profiles and insomnia in PD patients and (2) identify differences in this relationship between PD patients and non-PD individuals. By addressing these questions, our study aims to provide insights into the associations between the gut microbiome and insomnia in PD and to explore whether these relationships differ between PD patients and non-PD individuals, which may inform future research on microbiome-based approaches tailored to PD patients.

## Methods

### Participant recruitment and data collection

**Figure 1:**
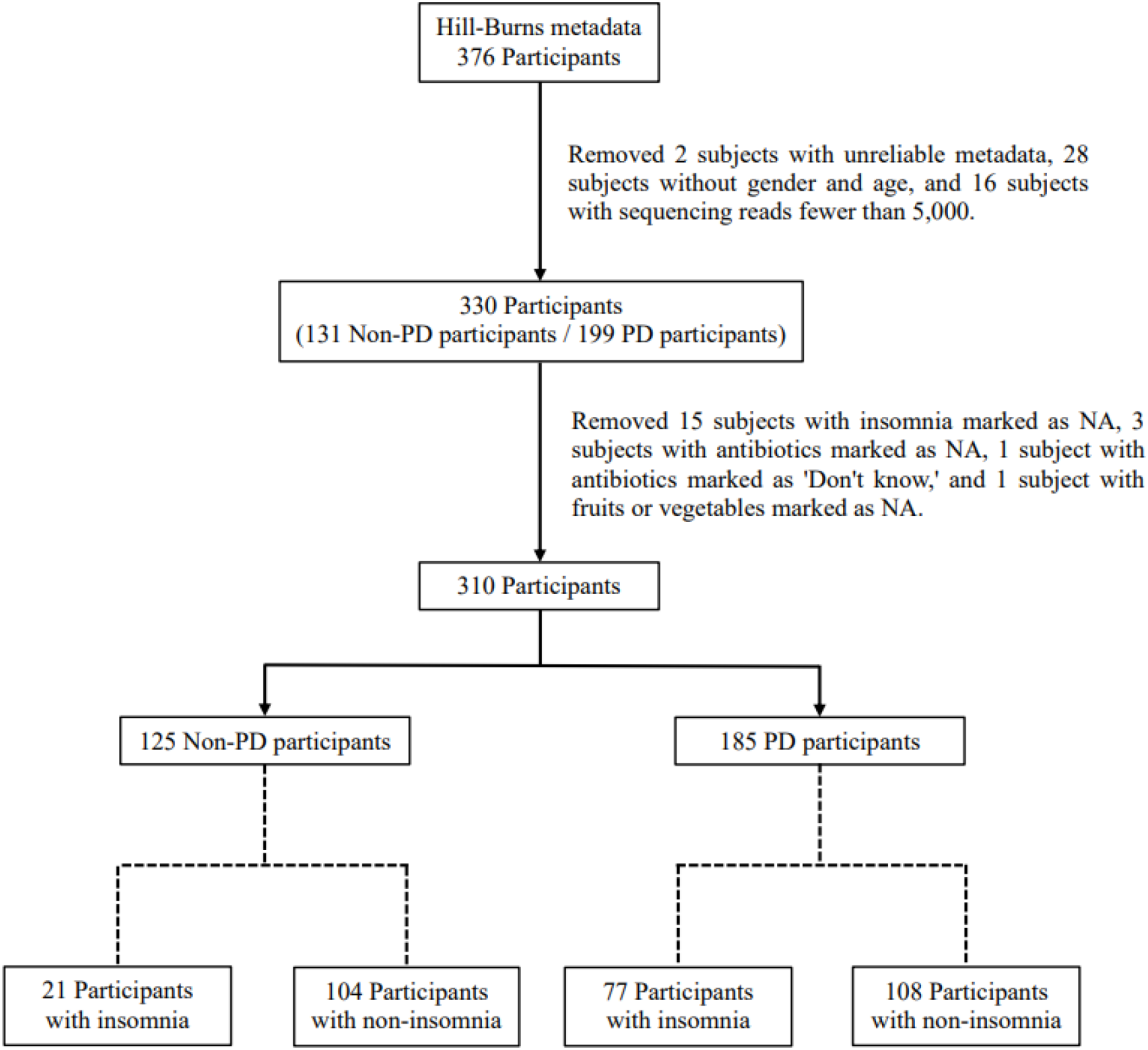
Participant selection flowchart. From 376 initial participants in Hill-Burns’ dataset, exclusions for missing data and low sequencing reads reduced the cohort to 310 (125 non-PD, 185 PD). Participants were then categorized into insomnia and non-insomnia groups for analysis.

We utilized and modified the data from the study of Hill-Burns et al (Hill-Burns et al., 2017), which involved 376 participants enrolled in the NeuroGenetics Research Consortium between March 2014 and January 2015. A total of 46 participants were excluded due to data quality issues: 2 participants had incomplete metadata (e.g., missing key demographic details), 28 participants lacked both gender and age information, and 16 participants had sequencing reads fewer than 5,000. The methods and the clinical and genetic characteristics of the NeuroGenetics Research Consortium dataset were described in detail by Hamza et al (Hamza et al., 2010). Of the remaining 330 participants, 199 (133 males, 66 females, mean age: 68.43 years) were diagnosed with PD based on the modified UK Brain Bank criteria (Clarke et al., 2016). The remaining 131 participants (52 males, 79 females, mean age: 70.37 years) self-reported as being free of neurodegenerative disease. The use insomnia medication was used as the proxy to assess insomnia symptoms. We excluded 15 subjects without insomnia status, 3 subjects without antibiotic status, 1 subject who reported an antibiotic status of “Don’t know”, and 1 subject without information on fruit or vegetable intake. The final analysis included 310 participants: 185 PD patients (126 males, 59 females, mean age: 68.01 years), and 125 controls (49 males, 76 females, mean age: 70.07 years). The flow chart of above inclusion and exclusion criteria are demonstrated in **Figure 1**. To evaluate potential bias due to sample exclusions, we compared the distributions of PD status, age, and gender between excluded and included participants in the final exclusion step. No significant differences were observed for PD status or gender, while age showed only a borderline difference (**Supplementary Table 2**). Detailed procedures for fecal sample collection, DNA extraction, sequencing, and metadata collection are described in Hill-Burns et al (Hill-Burns et al., 2017).

### Analysis of 16S rRNA sequence data

The 16S rRNA gene is highly conserved among bacteria, making it an ideal target for DNA sequencing in bacterial identification. The adapter sequences were removed using Trimmomatic v0.39 (Bolger et al., 2014). The resulting reads were subsequently processed, aligned, and denoised using DADA2 1.16 (Callahan et al., 2016). In brief, sequence reads were filtered using the recommended parameters in DADA2. Filtered reads were then de-replicated and de-noised using the default settings in DADA2. An amplicon sequence variant (ASV) table was constructed, and taxonomy assignment was performed using the SILVA v132 database, as natively implemented in DADA2. Species-level annotations were added using the addSpecies function in DADA2, with the SILVA database serving as the reference. Samples with fewer than 5,000 reads were excluded prior to rarefaction. To standardize sequencing depth across the remaining samples, rarefaction was then applied at a threshold of 5,000 reads per sample using the rarefy_even_depth function in phyloseq v1.32.0 (McMurdie and Holmes, 2013). For the subsequent statistical analysis, sequence counts were normalized to relative abundance by dividing the count of sequences assigned to each ASV by the total sequence count for the sample. Alpha and beta diversity indices were calculated from the rarefied ASV table prior to prevalence filtering. For differential abundance analyses and functional enrichment analysis, only ASVs present in at least 10% of samples were retained.

### Statistical analysis

We compared demographic characteristics - including age, sex, use of antibiotics or probiotics, and dietary factors (fruits, vegetables, grains, meats, nuts, and yogurt) - between insomnia and non-insomnia individuals in both the PD and non-PD groups. These comparisons were conducted using the Kruskal-Wallis test for continuous variables and the chi-square test for categorical variables. To evaluate overall taxonomic diversity, we calculated alpha and beta diversities between insomnia and non-insomnia groups. For alpha diversity, we estimated observed richness (i.e., number of ASVs), Chao1, Shannon, and Simpson indices from the ASV table (Chao, 1984; Rosenzweig, 1995; Magurran, 2013) using phyloseq 1.50.0 (McMurdie and Holmes, 2013). P-values for alpha diversity were obtained through analysis of variance using Stats 4.4.2. For beta diversity, we assessed the dissimilarities (distances) between groups using unweighted unique fraction metrics (Unifrac), weighted Unifrac (Lozupone et al., 2011), and Canberra distance (Lance and Williams, 1967), all calculated with phyloseq 1.50.0 (McMurdie and Holmes, 2013). P-values for beta diversity were calculated with ADONIS with Vegan 2.6.4. To evaluate the potential influence of sequencing depth, we additionally performed sensitivity analyses by including sequencing depth as a covariate in the statistical models. We also analyzed microbial differences in relative abundance between insomnia and non-insomnia individuals separately for both the PD and non-PD groups. These analyses were conducted using generalized linear models with a negative binomial distribution, which is commonly used for microbiome count data due to its ability to account for overdispersion. To reduce sparsity and excessive zero counts frequently observed in microbiome datasets, taxa present in fewer than 10% of samples were excluded prior to differential abundance analyses. Because all samples were rarefied to the same sequencing depth (5,000 reads per sample), sequencing depth was not included as an offset in the negative binomial models.

### Functional enrichment analysis of predicted metagenomes

We used Phylogenetic Investigation of Communities by Reconstruction of Unobserved States (PICRUSt2) version 2.4.1 (Douglas et al., 2020) to predict the functional potential of the gut microbiome in the samples, following the recommended pipeline of normalizing ASVs by copy number (to account for differences in number of copies of 16S rRNA between taxa), predicting functions using Kyoto Encyclopedia of Genes and Genomes (KEGG) (Kanehisa and Goto, 2000) orthologs, and grouping predicted pathways by KEGG hierarchical level 3. We compared metabolic pathway profiles between insomnia and non-insomnia individuals within the PD and non-PD groups using the Statistical Analysis of Metagenomic Profiles (STAMP) software version 2.1.3 (Parks and Beiko, 2013). White’s non-parametric t-test (two-sided, permutation = 1,000) was applied, and significance was determined using a Storey false discovery rate (FDR) < 0.05.

### Data availability and ethical statement

The sequences analyzed in this study are accessible at the European Nucleotide Archive (ENA) under the accession number ERP016332. All data are publicly accessible and anonymized. Ethical approval is not necessary.

## Results

### Demographic characteristics

**Figure 2:**
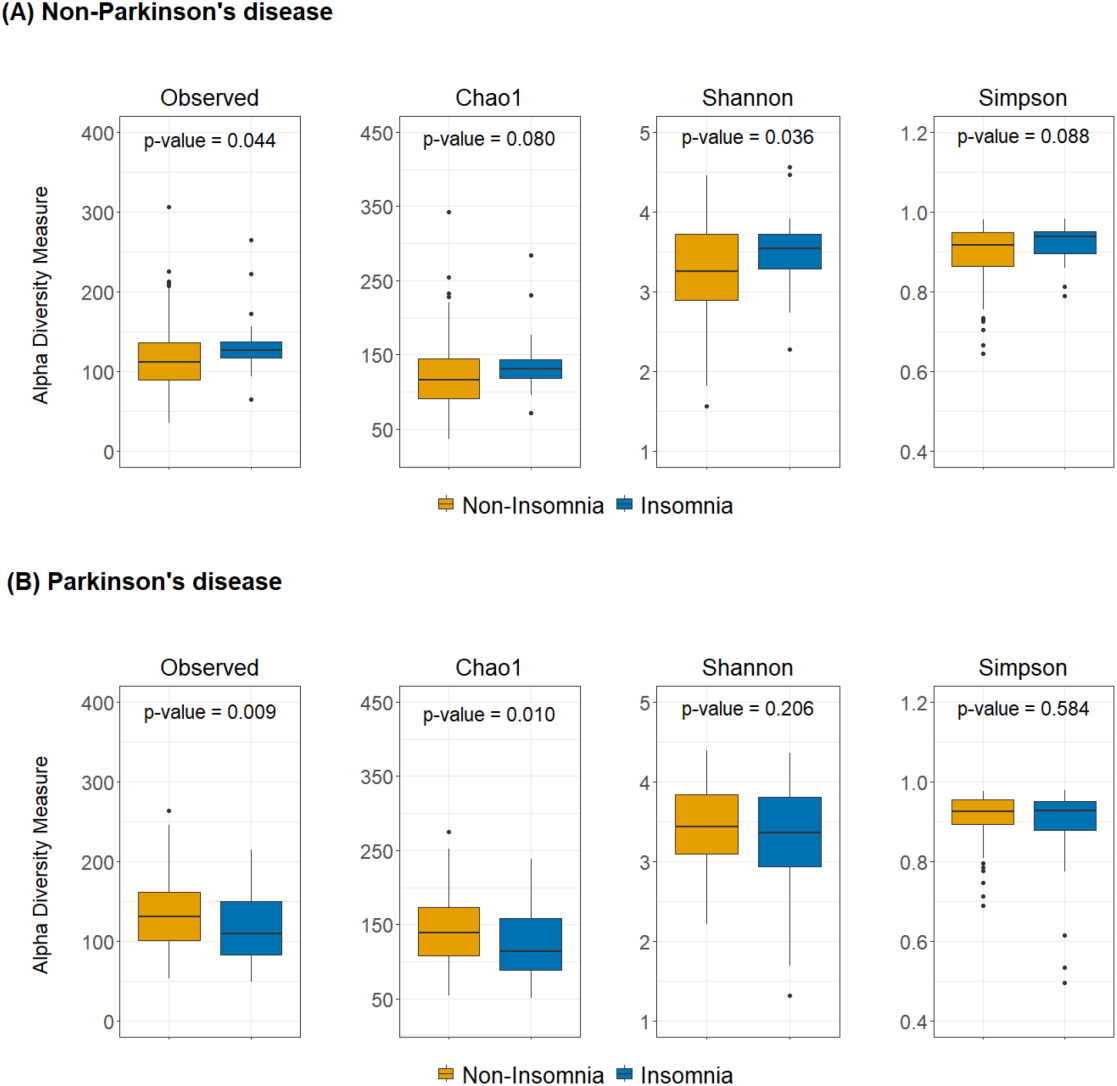
Alpha diversity differences between insomnia and non-insomnia groups in both **(A)** non-PD and **(B)** PD groups. The boxplots show the alpha diversity of the bacterial communities by means of observed amplicon sequence variants (ASVs), and Chao1, Shannon, and Simpson indexes. Median, and also lower and upper quartiles are shown on the plots.

**Figure 3:**
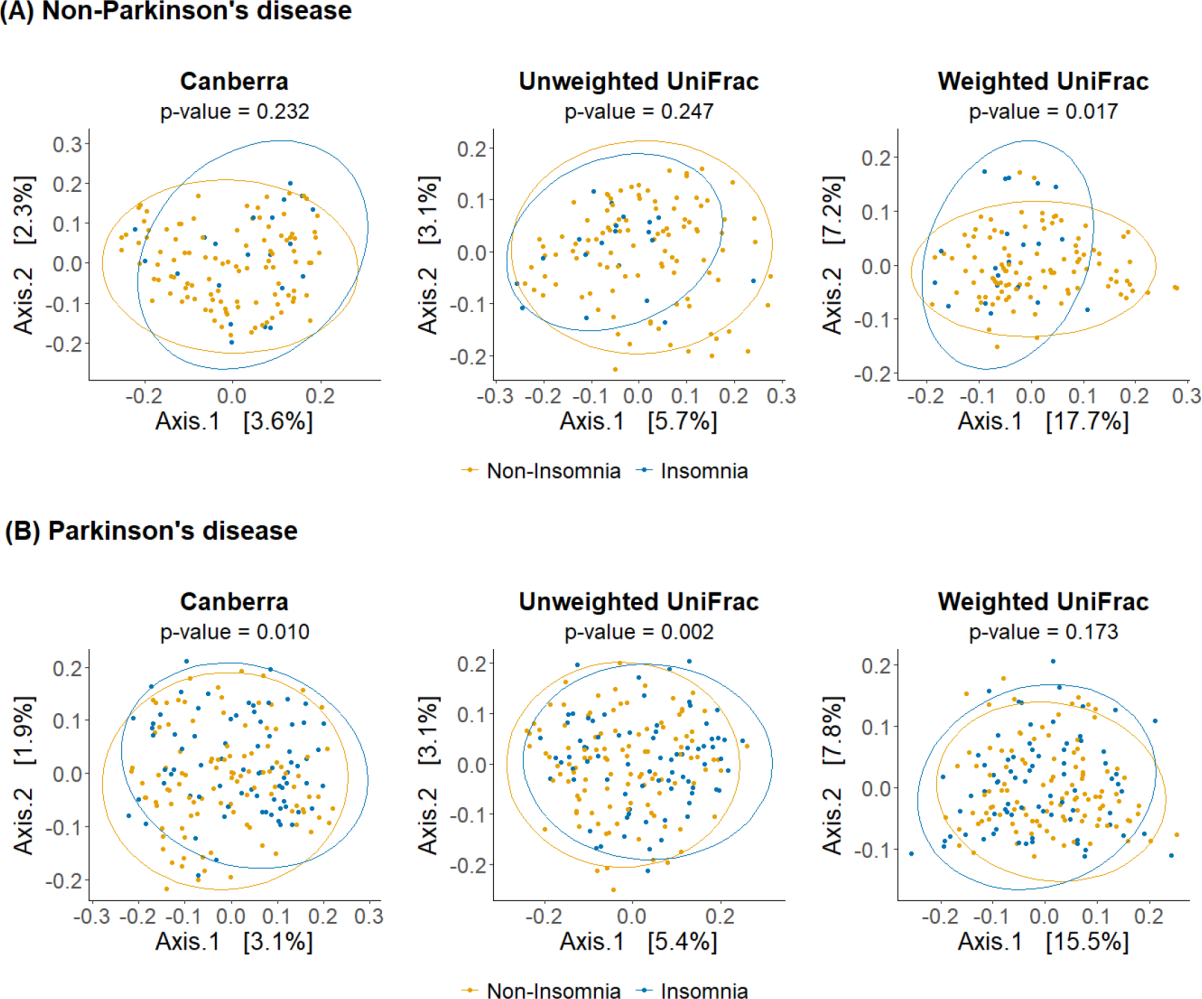
Beta diversity differences between insomnia and non-insomnia groups in both **(A)** non-PD and **(B)** PD groups. The PCoA plots show the following 3 distance measures: Canberra, unweighted unique fraction metric (UniFrac), and weighted UniFrac.

**Figure 4:**
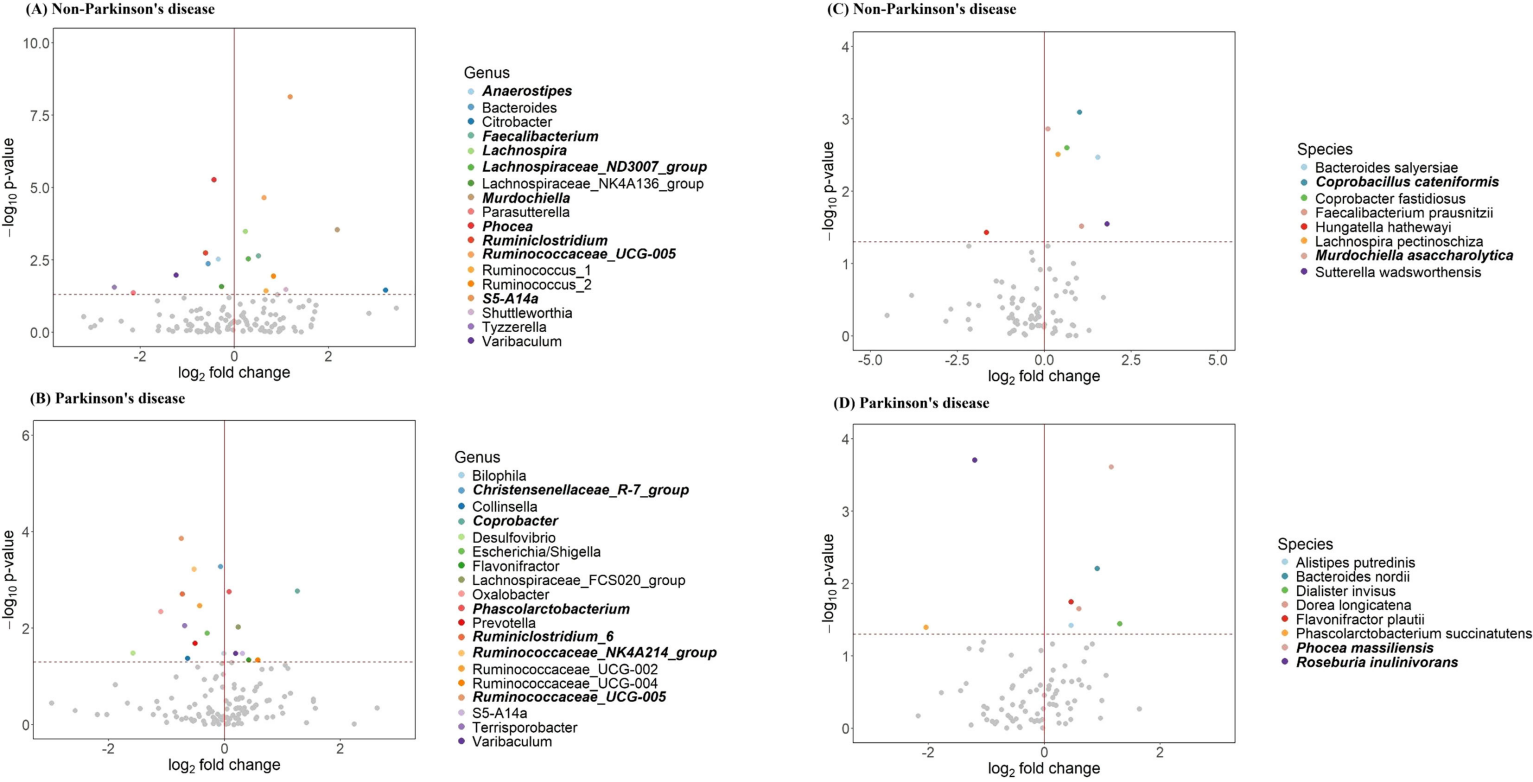
Differentially abundant taxa before FDR correction. Volcano plots show microbial taxa associated with insomnia in **(A)** non-PD and **(B)** PD groups at the genus level, and **(C)** non-PD and **(D)** PD groups at the species level. Each point represents a taxon, with log_2_ fold change on the x-axis and -log_10_ p-value on the y-axis. The red vertical line indicates no change, while the horizontal dashed line marks the significance threshold. Colored points denote significant taxa before FDR correction, and bolded names indicate significance after FDR correction.

**Figure 5:**
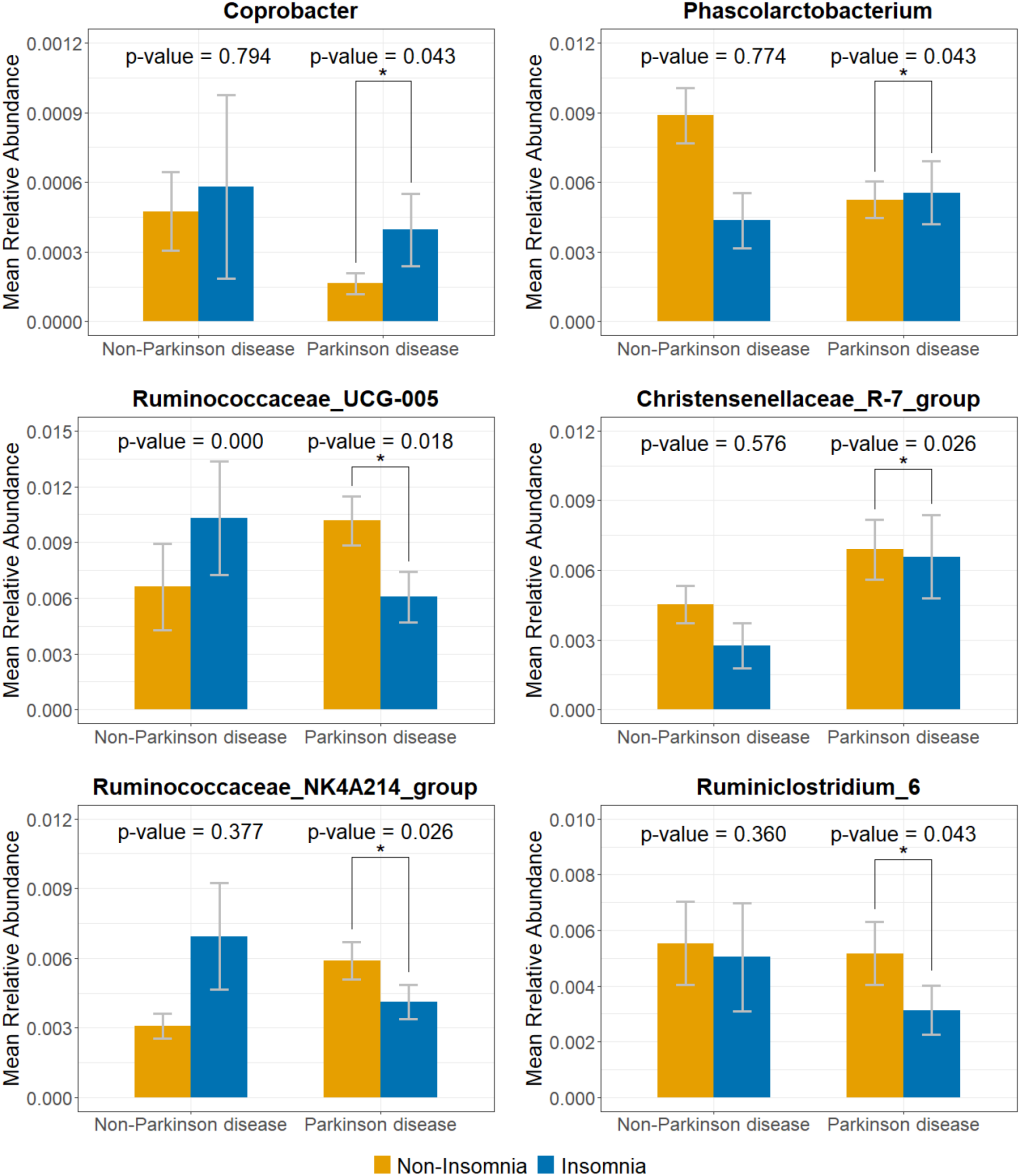
Relative abundance of insomnia-associated genera in PD and non-PD groups. Mean relative abundance of six insomnia-associated genera in both PD and non-PD groups, comparing insomnia and non-insomnia participants. Significant differences were observed in the PD group, but these genera were either non-significant or showed opposite trends in the control group. Error bars represent mean ± standard deviation. The symbol “*” indicates a statistically significant difference at the 0.05 significance level.

**Figure 6:**
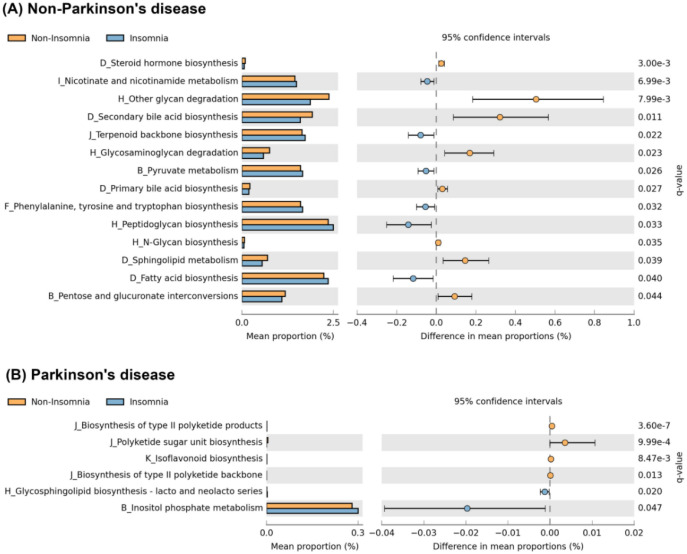
Functional pathway enrichment in insomnia participants. Functional pathways significantly enriched in insomnia participants differed between PD and non-PD groups. The control group showed enrichment in six pathways related to metabolism and biosynthesis, while the PD group exhibited enrichment in only two pathways linked to neuroinflammation and cell signaling. No overlapping pathways were observed between groups.

Participants were categorized into two groups: the PD group and the non-PD group. Within each group, participants were further divided into those with insomnia and those without insomnia. We compared demographic variables such as age, gender, antibiotics and probiotics consumptions, and dietary habits, including daily fruit or vegetable intake and grain consumption, as shown in **Table 1**. The analysis revealed no statistically significant differences in demographic characteristics, except age and antibiotics consumption, between the insomnia and non-insomnia groups within either the PD or non-PD group. However, factors such as gender and dietary habits (e.g., fruit and vegetable intake) are known to significantly influence the gut microbiome. Therefore, age, gender, dietary habits, and antibiotic use, regardless of the statistically significant differences, these variables were controlled as confounders in subsequent analyses to ensure robust results. To address potential confounding by PD-related clinical factors, we additionally evaluated the influence of PD medications, disease duration, and psychiatric comorbidities on the observed associations. Specifically, medication variables included levodopa/carbidopa, dopamine agonists (including pramipexole, ropinirole, and rotigotine), monoamine oxidase B inhibitors (selegiline and rasagiline), catechol-O-methyltransferase inhibitors (entacapone and tolcapone), amantadine, apomorphine, and anticholinergic agents. PD duration and several psychiatric comorbidities, including depression, bipolar disorder, anorexia, and phobia, were also examined (**Supplementary Table 1**). Among these variables, only depression showed a significant association with insomnia status, whereas PD medications, disease duration, and other psychiatric conditions were not significantly associated with insomnia in this cohort. To further evaluate the potential confounding effect of depression, we repeated the primary analyses with additional adjustment for depression. The results remained largely unchanged, and the overall patterns observed in **Figures 2**–**6** were consistent with the primary analyses (**Supplementary Figures S2**-**S6**).

**Table 1:** Comparing Parkinson’s disease (PD) patients and healthy individuals (Control) with and without insomnia on demographic variables.

Variables	Non-Parkinson’s disease			Parkinson’s disease		
	Insomnia (N = 21)	W/o insomnia (N = 104)	P-value	Insomnia (N = 77)	W/o insomnia (N = 108)	P-value
**Gender**			0.266			0.207
Female	10 (47.6%)	66 (63.5%)		29 (37.7%)	30 (27.8%)	
Male	11 (52.4%)	38 (36.5%)		48 (62.3%)	78 (72.2%)	
**Age**			0.205			0.006
≦ 65	3 (14.3%)	32 (30.8%)		39 (50.6%)	32 (29.6%)	
> 65	18 (85.7%)	72 (69.2%)		38 (49.4%)	76 (70.4%)	
**Antibiotics**			0.995			0.009
No	21 (100.0%)	101 (97.1%)		69 (89.6%)	107 (99.1%)	
Yes	0 (0.0%)	3 (2.9%)		8 (10.4%)	1 (0.9%)	
**Probiotics**			0.684			0.424
No	0 (0.0%)	73 (70.2%)		53 (68.8%)	81 (75.0%)	
Yes	16 (76.2%)	28 (26.9%)		18 (23.4%)	23 (21.3%)	
Missing	0 (0.0%)	3 (2.9%)		6 (7.8%)	4 (3.7%)	
**Eat fruits or vegetable daily**			0.471			0.957
No	4 (19.0%)	11 (10.6%)		16 (20.8%)	24 (22.2%)	
Yes	17 (81.0%)	93 (89.4%)		61 (79.2%)	84 (77.8%)	
**Eat grains daily**			0.822			0.454
No	8 (38.1%)	34 (32.7%)		22 (28.6%)	33 (30.6%)	
Yes	13 (61.9%)	70 (67.3%)		55 (71.4%)	73 (67.6%)	
Missing	0 (0.0%)	0 (0.0%)		0 (0.0%)	2 (1.9%)	
**Eat meats daily**			1.000			0.572
No	13 (61.9%)	40 (38.5%)		36 (46.8%)	45 (41.7%)	
Yes	17 (81.0%)	64 (61.5%)		41 (53.2%)	62 (57.4%)	
Missing	0 (0.0%)	0 (0.0%)		0 (0.0%)	2 (0.9%)	
**Eat nuts daily**			0.488			0.502
No	17 (81.0%)	71 (68.3%)		58 (75.3%)	87 (80.6%)	
Yes	4 (19.0%)	32 (30.8%)		19 (24.7%)	21 (19.4%)	
Missing	0 (0.0%)	1 (1.0%)		0 (0.0%)	0 (0.0%)	
**Eat yogurt daily**			0.218			0.933
No	21 (100.0%)	92 (88.5%)		71 (92.2%)	98 (90.7%)	
Yes	0 (0.0%)	12 (11.5%)		6 (7.8%)	10 (9.3%)	

### Contrasting gut microbiome diversity patterns in PD and non-PD groups

To investigate whether insomnia affects gut microbiome diversity differently in PD and non-PD groups, we analyzed alpha and beta diversity metrics between participants with and without insomnia. **Figure 2** illustrates the differences in gut microbiome alpha diversity between participants with and without insomnia in both groups. In the non-PD group, participants with insomnia exhibited significantly higher alpha diversity compared to those without insomnia, as demonstrated by the Observed and Shannon indices (p = 0.044 and 0.036, respectively; **Figure 2A**). Conversely, in the PD group, participants with insomnia had significantly lower alpha diversity compared to non-insomnia participants, with the Observed and Chao1 indices reaching statistical significance (p = 0.009 and 0.010, respectively; **Figure 2B**). These contrasting patterns suggest that the impact of insomnia on alpha diversity differs between the non-PD and PD groups. To further evaluate these differences, an interaction analysis was performed, revealing significant interactions between insomnia status and group type (PD vs. control) for the Observed, Chao1, and Shannon indices (p = 0.002, 0.004, and 0.015, respectively; data not shown in **Figure 2**). Interaction analysis examines whether the effect of one variable (e.g., insomnia status) on an outcome (e.g., diversity indices) differs depending on the levels of another variable (e.g., group type). These results indicate that the mechanisms driving insomnia may differ between PD patients and the general population, a topic further addressed in the Discussion section.

**Figure 3** presents beta diversity analyses, highlighting significant differences between participants with and without insomnia in both groups. In the non-PD group, weighted UniFrac distance differed significantly between insomnia and non-insomnia individuals (p = 0.017; **Figure 3A**). In the PD group, significant differences were observed in unweighted UniFrac distance (p = 0.002) and Canberra distance (p = 0.010) between the two groups (**Figure 3B**). These findings also suggest that the effect of insomnia on beta diversity varies between non-PD and PD groups. To explore this further, an interaction analysis was conducted, showing significant interactions between insomnia status and PD status across all three beta diversity metrics: Canberra, Unweighted UniFrac, and Weighted UniFrac (p = 0.010, 0.005, and 0.002, respectively; data not shown in **Figure 3**).

### Bacterial genera and species associated with insomnia in PD and non-PD individuals

To identify gut bacterial taxa associated with insomnia in both PD and non-PD individuals, we compared the relative abundance of microbial genera between insomnia and non-insomnia groups, as demonstrated by the volcano plots in **Figure 4**. To minimize inflated type I error (false discovery rate, FDR) due to multiple testing, we applied the Benjamini-Hochberg FDR correction. After FDR correction, 6 and 7 bacterial genera out of 132 were found to be significantly associated with insomnia in both PD and non-PD groups. Among PD patients, insomnia-risk-reducing taxa included, *Ruminococcaceae_UCG-005, Christensenellaceae_R-7_group, Ruminococcaceae_NK4A214_group*, and *Ruminiclostridium_6*, whereas insomnia-risk-increasing taxa comprised *Coprobacter* and *Phascolarctobacterium*. In the non-PD group, insomnia-risk-reducing taxa was *Bacteroides*, while insomnia-risk-increasing taxa included *Ruminococcaceae_UCG-005, Lachnospira, Faecalibacterium, Lachnospiraceae_ND3007_group, Coprobacillus*, and *Murdochiella*. Interestingly, among the six bacterial genera significantly associated with insomnia in the PD group, their relative abundance patterns differed notably in the control group, shown in **Figure 5**. Specifically, while genera such as *Coprobacter* and *Phascolarctobacterium* were significantly enriched in insomnia cases within the PD group, their differences were non-significant in the control group. Conversely, *Ruminococcaceae_UCG-005*, which showed a significant decrease in insomnia cases within the PD group, exhibited a contrasting pattern in the control group. At the species level, in the non-PD group, insomnia was significantly associated with the relative abundance of *Coprobacillus cateniformis* and *Murdochiella asaccharolytica*, both of which exhibited a positive association with insomnia. Conversely, in the PD group, insomnia was linked to an increase in *Phocea massiliensis*). Additionally, *Roseburia inulinivorans* was negatively associated with insomnia in the PD group, suggesting that its reduced abundance may be a potential marker for insomnia in PD patients. These findings highlight distinct gut microbial associations with insomnia in PD patients compared to non-PD individuals, indicating potential differences in the underlying mechanisms driving insomnia in these populations.

### Predicted functional pathways associated with insomnia in PD and non-PD individuals

The results of the functional analysis are presented in **Figure 6**. In the non-PD group, six functional pathways were enriched among participants with insomnia: (1) nicotinate and nicotinamide metabolism, (2) terpenoid backbone biosynthesis, (3) pyruvate metabolism, (4) phenylalanine, tyrosine and tryptophan biosynthesis, (5) peptidoglycan biosynthesis, and (6) fatty acid biosynthesis. In contrast, in the PD group, only two functional pathways were enriched among participants with insomnia: (1) glycosphingolipid biosynthesis - lacto and neolacto series, and (2) inositol phosphate metabolism. Notably, there was no overlap in the enriched functional pathways between the two groups. These findings align with the observed differences in alpha and beta diversity and microbial relative abundance, further supporting distinct microbiome-associated functional profiles linked to insomnia in PD patients compared to non-PD individuals.

## Discussion

This study provides the first comprehensive analysis of the association between insomnia and gut microbiome profiles in both PD patients and non-PD individuals. Our findings revealed distinct differences between the two groups in alpha and beta diversity, key bacterial genera and species, and predicted functional pathways associated with insomnia. These results suggest that microbiome-based therapeutic approaches for managing insomnia may need to consider the specific context of PD rather than relying solely on general strategies. In the following sections, we discuss these findings in detail, focusing on microbial diversity, taxonomic composition, and predicted functional pathways.

Regarding microbial diversity, insomnia showed contrasting associations with alpha diversity in PD patients and non-PD individuals. In the non-PD group, participants with insomnia exhibited significantly higher alpha diversity, while those in the PD group showed significantly lower alpha diversity. To our knowledge, no previous studies have specifically examined the relationship between insomnia and the gut microbiome in PD patients, making our findings novel in this context. The closest comparable study, conducted by Tanaka et al., investigated insomnia in individuals with depression and/or anxiety and similarly reported decreased alpha diversity in the insomnia group (Tanaka et al., 2023). This alignment suggests that the associations between insomnia and microbial diversity observed in PD may share similarities with those reported in other neuropsychiatric conditions. In contrast, Jiang et al., a large-scale study conducted in China, found reduced alpha diversity in patients with new-onset or chronic insomnia, a finding opposite to our observations in the non-PD group (Jiang et al., 2022). This discrepancy could be attributed to differences in genetic background and dietary habits, as our study focused on a predominantly Caucasian population, whereas Jiang et al. examined a Chinese cohort (Jiang et al., 2022). These population-specific differences highlight the need for further research to better understand the role of genetic and environmental factors in shaping microbial diversity in insomnia. On the other hand, the significant beta diversity differences observed in our study align with the findings from Jiang et al., which demonstrated significant beta diversity changes among insomnia subgroups (i.e. new-onset insomnia vs. chronic insomnia) (Jiang et al., 2022), further reinforcing the importance of disease context in interpreting microbial diversity.

At the taxonomic level, several bacterial taxa showed distinct associations with insomnia in PD patients and non-PD individuals. Among PD patients, insomnia was positively associated with *Coprobacter* and *Phascolarctobacterium* and negatively associated with *Ruminococcaceae* and *Christensenellaceae*. In contrast, insomnia in non-PD individuals was positively associated with *Ruminococcaceae*, *Lachnospira*, *Faecalibacterium, Lachnospiraceae*, *Coprobacillus*, and *Murdochiella*, while *Bacteroides* was negatively associated. These findings partially align with previous studies, such as Yan et al (Yan et al., 2024), which identified *Coprobacter* as potentially protective against sleep disturbances and *Lachnospiraceae* as a risk factor. Although our finding regarding *Coprobacter* contrasts with Yan et al.’s report, their study was conducted on a younger general population (mean age: 44.3 years) that excluded individuals with neurological and mental disorders, making differences in the findings between their cohort and PD patients unsurprising.

Regarding *Bacteroidetes* levels, we observed a decrease in the insomnia group within our non-PD individuals, consistent with findings from Yang et al (Yang et al., 2021). and Zhou et al (Zhou et al., 2022), who also reported reduced *Bacteroidetes* levels in individuals with insomnia. However, this contrasts with Tanaka et al., which found elevated *Bacteroidetes* levels associated with insomnia (Tanaka et al., 2023). These discrepancies may be due to differences in the study populations, as Tanaka’s study focused on individuals with depression and anxiety rather than individuals without these conditions. Although Bacteroides did not show a statistically significant association with insomnia in the PD group after FDR correction, the interaction analysis between PD status and insomnia revealed a significant difference in microbial relative abundance. This finding supports the central argument of our study—namely, that the relationship between insomnia and the gut microbiome in PD patients differs from that observed in non-PD individuals.

Our study identified distinct microbiome-associated functional pathways related to insomnia in PD patients compared with non-PD individuals. In the non-PD group, six functional pathways were enriched, including nicotinate and nicotinamide metabolism, terpenoid backbone biosynthesis, pyruvate metabolism, phenylalanine, tyrosine, and tryptophan biosynthesis, peptidoglycan biosynthesis, and fatty acid biosynthesis. These pathways are closely linked to energy production, vitamin metabolism, and the synthesis of neurotransmitter precursors, all of which are related to maintaining normal sleep-wake cycles (Irmisch et al., 2007; Malik et al., 2024). For example, nicotinate and nicotinamide metabolism plays an essential role in NAD+ production, which is directly tied to cellular energy balance and circadian rhythm regulation (Xie et al., 2020; Basse et al., 2023).

In contrast to non-PD group, PD patients with insomnia exhibited enrichment in only two functional pathways: glycosphingolipid biosynthesis (lacto and neolacto series) and inositol phosphate metabolism, both of which are primarily involved in cell membrane structure and intracellular signaling. Glycosphingolipids are key structural components of neuronal membranes (Belarbi et al., 2020), and disruptions in their biosynthesis have been associated with neurodegenerative processes and inflammatory responses. Meanwhile, inositol phosphate metabolism plays a crucial role in signal transduction and calcium homeostasis (Hughes and Putney, 1990; Onu et al., 2025), both of which are vital for maintaining synaptic function. The findings of enrichment in the two functional pathway reinforce the role of neuroinflammation in PD-related sleep disturbances, as evidence suggests that peripheral T cell infiltration and glial cell activation contribute to neurodegeneration in α-synucleinopathies (Iba et al., 2020). Additionally, sleep disturbances have been linked to abnormal cerebrospinal fluid alpha-synuclein levels, suggesting an association between poor sleep and PD pathology (Wang et al., 2020). The lack of overlapping enriched pathways suggests that microbiome-associated functional profiles related to insomnia differ between PD patients and non-PD individuals. In PD patients, pathways related to neuroinflammation and neurodegeneration were more frequently enriched, whereas metabolically related pathways were more commonly observed in non-PD individuals. However, given the cross-sectional design and the predictive nature of the functional analysis, these results should be interpreted as hypothesis-generating rather than evidence of causal biological mechanisms. Nevertheless, these findings may provide insights for future studies investigating microbiome-related processes involved in sleep disturbances in PD.

Despite the important insights provided by this study, several limitations should be acknowledged. First, insomnia in this study was operationalized using the reported use of sleep medication as a proxy measure. This approach may not capture all individuals experiencing insomnia symptoms and may introduce exposure misclassification. Therefore, the proxy measure may reflect both insomnia symptoms and treatment-related behaviors rather than a clinically validated insomnia diagnosis. Future studies with detailed sleep assessments and clinical measurements would help clarify these relationships. In addition, it is worth noting that if this exposure misclassification is non-differential, the estimated associations may be biased toward the null, potentially reducing the ability to detect true associations (Rothman et al., 2008).

Second, this study used a cross-sectional design, which limits causal inference. The temporal relationship between gut microbiome alterations and insomnia cannot be determined from the present data. Several alternative causal interpretations are possible. For example, gut microbiome alterations may contribute to insomnia, whereas insomnia or sleep-related behaviors may influence gut microbiome composition. Because insomnia was defined using a proxy measure based on sleep medication use, some of the observed associations may also reflect medication-related effects on the gut microbiome. Under certain causal structures, sleep medication use may additionally lie on the pathway between insomnia and gut microbiome alterations, potentially acting as a mediator. Therefore, the exposure definition may capture both insomnia symptoms and related treatment behaviors.

Third, we relied on fecal samples to represent gut microbiome composition, which, while standard practice, may not fully capture microbial variations across different gut regions. Recent research has shown substantial differences in bacterial composition between the small and large intestines, suggesting that sampling from multiple gut regions could enhance understanding of microbiome contributions to insomnia (Jensen et al., 2023; Li et al., 2023). Fourth, while rarefaction was employed to standardize sequencing depth as a commonly used approach in microbiome analyses (Chiarello et al., 2022; Hong et al., 2022; Schloss, 2024), we recognize that alternative normalization approaches exist. Sensitivity analyses incorporating sequencing depth as a covariate yielded consistent results, supporting the robustness of our findings (**Supplementary Figure 1**). Finally, the sample size, although adequate for initial exploration, may limit the generalizability of the findings.

In conclusion, our findings suggest that the gut microbiome profiles associated with insomnia differ between PD patients and non-PD individuals. While gut–brain interactions are known to play an important role in sleep regulation, the observed patterns in this study indicate that insomnia in PD may be associated with microbiome-related processes linked to neuroinflammation and neurodegeneration, whereas metabolic pathways may play a more prominent role in non-PD individuals. These observations highlight the importance of considering disease context when investigating microbiome–sleep relationships. Future studies should further investigate how microbiome-associated pathways contribute to sleep disturbances in PD and whether microbiome-based approaches may need to be tailored to the specific characteristics of PD patients. Longitudinal studies with larger sample sizes and more comprehensive data collection will be essential to clarify the directionality of these associations and to better understand the microbiome-related processes involved in insomnia in PD.

## Data Availability

Publicly available datasets were analyzed in this study. This data can be found here: The datasets analyzed for this study are publicly available in the European Nucleotide Archive (ENA) under accession number ERP016332: https://www.ebi.ac.uk/ena/browser/view/ERP016332.
