# Supplementary material for: Heterogeneity in the association between gut microbiota and insomnia moderated by Parkinson’s disease status
Source: Front Cell Infect Microbiol. 2026 May 5;16:1691665. doi: 10.3389/fcimb.2026.1691665 (PMC13183661; doi:10.3389/fcimb.2026.1691665)
Supplement: Supplementary file 1 [file Table1.docx]

**Supplementary of “Heterogeneity in the Association Between Gut Microbiota and Insomnia Moderated by Parkinson’s Disease Status”**

**Table S1.** Comparing Parkinson’s disease (PD) and non-PD individuals with and without insomnia on demographic variables.

|  | **Non-Parkinson's disease** | | | **Parkinson's disease** | | |
| --- | --- | --- | --- | --- | --- | --- |
|  | **Insomnia (N = 21)** | **w/o Insomnia (N = 104)** | **p-value** | **Insomnia (N = 77)** | **w/o Insomnia (N = 108)** | **p-value** |
| **Gender** |  |  | 0.266 |  |  | 0.207 |
| Female | 10 (47.6%) | 66 (63.5%) |  | 29 (37.7%) | 30 (27.8%) |  |
| Male | 11 (52.4%) | 38 (36.5%) |  | 48 (62.3%) | 78 (72.2%) |  |
| **Age** |  |  | 0.205 |  |  | 0.006 |
| ≦ 65 | 3 (14.3%) | 32 (30.8%) |  | 39 (50.6%) | 32 (29.6%) |  |
| ＞ 65 | 18 (85.7%) | 72 (69.2%) |  | 38 (49.4%) | 76 (70.4%) |  |
| **Antibiotics** |  |  | 0.995 |  |  | 0.009 |
| No | 21 (100.0%) | 101 (97.1%) |  | 69 (89.6%) | 107 (99.1%) |  |
| Yes | 0 (0.0%) | 3 (2.9%) |  | 8 (10.4%) | 1 (0.9%) |  |
| **Probiotics** |  |  | 0.684 |  |  | 0.424 |
| No | 0 (0.0%) | 73 (70.2%) |  | 53 (68.8%) | 81 (75.0%) |  |
| Yes | 16 (76.2%) | 28 (26.9%) |  | 18 (23.4%) | 23 (21.3%) |  |
| Missing | 0 (0.0%) | 3 (2.9%) |  | 6 (7.8%) | 4 (3.7%) |  |
| **Eat fruits or vegetable daily** |  |  | 0.471 |  |  | 0.957 |
| No | 4 (19.0%) | 11 (10.6%) |  | 16 (20.8%) | 24 (22.2%) |  |
| Yes | 17 (81.0%) | 93 (89.4%) |  | 61 (79.2%) | 84 (77.8%) |  |
| **Eat grains daily** |  |  | 0.822 |  |  | 0.454 |
| No | 8 (38.1%) | 34 (32.7%) |  | 22 (28.6%) | 33 (30.6%) |  |
| Yes | 13 (61.9%) | 70 (67.3%) |  | 55 (71.4%) | 73 (67.6%) |  |
| Missing | 0 (0.0%) | 0 (0.0%) |  | 0 (0.0%) | 2 (1.9%) |  |
| **Eat meats daily** |  |  | 1.000 |  |  | 0.572 |
| No | 13 (61.9%) | 40 (38.5%) |  | 36 (46.8%) | 45 (41.7%) |  |
| Yes | 17 (81.0%) | 64 (61.5%) |  | 41 (53.2%) | 62 (57.4%) |  |
| Missing | 0 (0.0%) | 0 (0.0%) |  | 0 (0.0%) | 2 (0.9%) |  |
| **Eat nuts daily** |  |  | 0.488 |  |  | 0.502 |
| No | 17 (81.0%) | 71 (68.3%) |  | 58 (75.3%) | 87 (80.6%) |  |
| Yes | 4 (19.0%) | 32 (30.8%) |  | 19 (24.7%) | 21 (19.4%) |  |
| Missing | 0 (0.0%) | 1 (1.0%) |  | 0 (0.0%) | 0 (0.0%) |  |
| **Eat yogurt daily** |  |  | 0.218 |  |  | 0.933 |
| No | 21 (100.0%) | 92 (88.5%) |  | 71 (92.2%) | 98 (90.7%) |  |
| Yes | 0 (0.0%) | 12 (11.5%) |  | 6 (7.8%) | 10 (9.3%) |  |
| **Depression** |  |  | 0.194 |  |  | <0.001 |
| No | 13 (61.9%) | 82 (78.8%) |  | 38 (49.4%) | 84 (77.8%) |  |
| Yes | 8 (38.1%) | 21 (20.2%) |  | 39 (50.6%) | 20 (18.5%) |  |
| Missing | 0 (0.0%) | 1 (1.0%) |  | 0 (0.0%) | 4 (3.7%) |  |
| **Bipolar** |  |  | 0.755 |  |  | 0.865 |
| No | 20 (95.2%) | 103 (99.0%) |  | 76 (98.7%) | 108 (100.0%) |  |
| Yes | 1 (4.8%) | 1 (1.0%) |  | 1 (1.3%) | 0 (0.0%) |  |
| **Anorexia** |  |  | NA |  |  | 0.865 |
| No | 21 (100%) | 104 (100%) |  | 76 (98.7%) | 108 (100.0%) |  |
| Yes |  |  |  | 1 (1.3%) | 0 (0.0%) |  |
| **Phobia** |  |  | 0.755 |  |  | 0.330 |
| No | 20 (95.2%) | 103 (99.0%) |  | 74 (96.1%) | 107 (99.1%) |  |
| Yes | 1 (4.8%) | 1 (1.0%) |  | 2 (2.6%) | 1 (0.9%) |  |
| Missing |  |  |  | 1 (1.3%) | 0 (0.0%) |  |
| **Carbidopa levodopa** |  |  |  |  |  | 0.592 |
| No |  |  |  | 9 (11.7%) | 8 (7.4%) |  |
| Yes |  |  |  | 64 (83.1%) | 95 (88.0%) |  |
| Missing |  |  |  | 4 (5.2%) | 5 (4.6%) |  |
| **Dopamine agonist** |  |  |  |  |  | 0.084 |
| No |  |  |  | 28 (36.4%) | 57 (52.8%) |  |
| Yes |  |  |  | 45 (58.4%) | 46 (42.6%) |  |
| Missing |  |  |  | 4 (5.2%) | 5 (4.6%) |  |
| **Pramipexole** |  |  |  |  |  | 0.959 |
| No |  |  |  | 52 (67.5%) | 75 (69.4%) |  |
| Yes |  |  |  | 21 (27.3%) | 28 (25.9%) |  |
| Missing |  |  |  | 4 (5.2%) | 5 (4.6%) |  |
| **Ropinirole** |  |  |  |  |  | 0.099 |
| No |  |  |  | 51 (66.2%) | 86 (79.6%) |  |
| Yes |  |  |  | 22 (28.6%) | 17 (15.7%) |  |
| Missing |  |  |  | 4 (5.2%) | 5 (4.6%) |  |
| **Rotigitine** |  |  |  |  |  | 0.661 |
| No |  |  |  | 71 (92.2%) | 102 (94.4%) |  |
| Yes |  |  |  | 2 (2.6%) | 1 (0.9%) |  |
| Missing |  |  |  | 4 (5.2%) | 5 (4.6%) |  |
| **Selegiline** |  |  |  |  |  | 0.873 |
| No |  |  |  | 65 (84.4%) | 94 (87.0%) |  |
| Yes |  |  |  | 8 (10.4%) | 9 (8.3%) |  |
| Missing |  |  |  | 4 (5.2%) | 5 (4.6%) |  |
| **Rasagiline** |  |  |  |  |  | 0.983 |
| No |  |  |  | 52 (67.5%) | 73 (67.6%) |  |
| Yes |  |  |  | 21 (27.3%) | 30 (27.8%) |  |
| Missing |  |  |  | 4 (5.2%) | 5 (4.6%) |  |
| **Mao b inhibitor** |  |  |  |  |  | 0.954 |
| No |  |  |  | 44 (57.1%) | 64 (59.3%) |  |
| Yes |  |  |  | 29 (37.7%) | 39 (36.1%) |  |
| Missing |  |  |  | 4 (5.2%) | 5 (4.6%) |  |
| **Entacapone** |  |  |  |  |  | 0.746 |
| No |  |  |  | 62 (80.5%) | 83 (76.9%) |  |
| Yes |  |  |  | 11 (14.3%) | 20 (18.5%) |  |
| Missing |  |  |  | 4 (5.2%) | 5 (4.6%) |  |
| **Tolcapone** |  |  |  |  |  | 0.481 |
| No |  |  |  | 73 (94.8%) | 101 (93.5%) |  |
| Yes |  |  |  | 0 (0.0%) | 2 (1.9%) |  |
| Missing |  |  |  | 4 (5.2%) | 5 (4.6%) |  |
| **Comt inhibitor** |  |  |  |  |  | 0.566 |
| No |  |  |  | 62 (80.5%) | 81 (75.0%) |  |
| Yes |  |  |  | 11 (14.3%) | 22 (20.4%) |  |
| Missing |  |  |  | 4 (5.2%) | 5 (4.6%) |  |
| **Amantadine** |  |  |  |  |  | 0.206 |
| No |  |  |  | 59 (76.6%) | 71 (65.7%) |  |
| Yes |  |  |  | 14 (18.2%) | 32 (29.6%) |  |
| Missing |  |  |  | 4 (5.2%) | 5 (4.6%) |  |
| **Apomorphine** |  |  |  |  |  | 0.944 |
| No |  |  |  | 72 (93.5%) | 101 (93.5%) |  |
| Yes |  |  |  | 1 (1.3%) | 2 (1.9%) |  |
| Missing |  |  |  | 4 (5.2%) | 5 (4.6%) |  |
| **Trihexiphenidylanticholinergic** |  |  |  |  |  | 0.075 |
| No |  |  |  | 73 (94.8%) | 96 (88.9%) |  |
| Yes |  |  |  | 0 (0.0%) | 7 (6.5%) |  |
| Missing |  |  |  | 4 (5.2%) | 5 (4.6%) |  |
| **Anticholinergic** |  |  |  |  |  | 0.075 |
| No |  |  |  | 73 (94.8%) | 96 (88.9%) |  |
| Yes |  |  |  | 0 (0.0%) | 7 (6.5%) |  |
| Missing |  |  |  | 4 (5.2%) | 5 (4.6%) |  |
| **Duration of PD** |  |  |  |  |  | 0.064 |
| Mean (SD) |  |  |  | 12.5 (6.6) | 14.3 (6.3) |  |


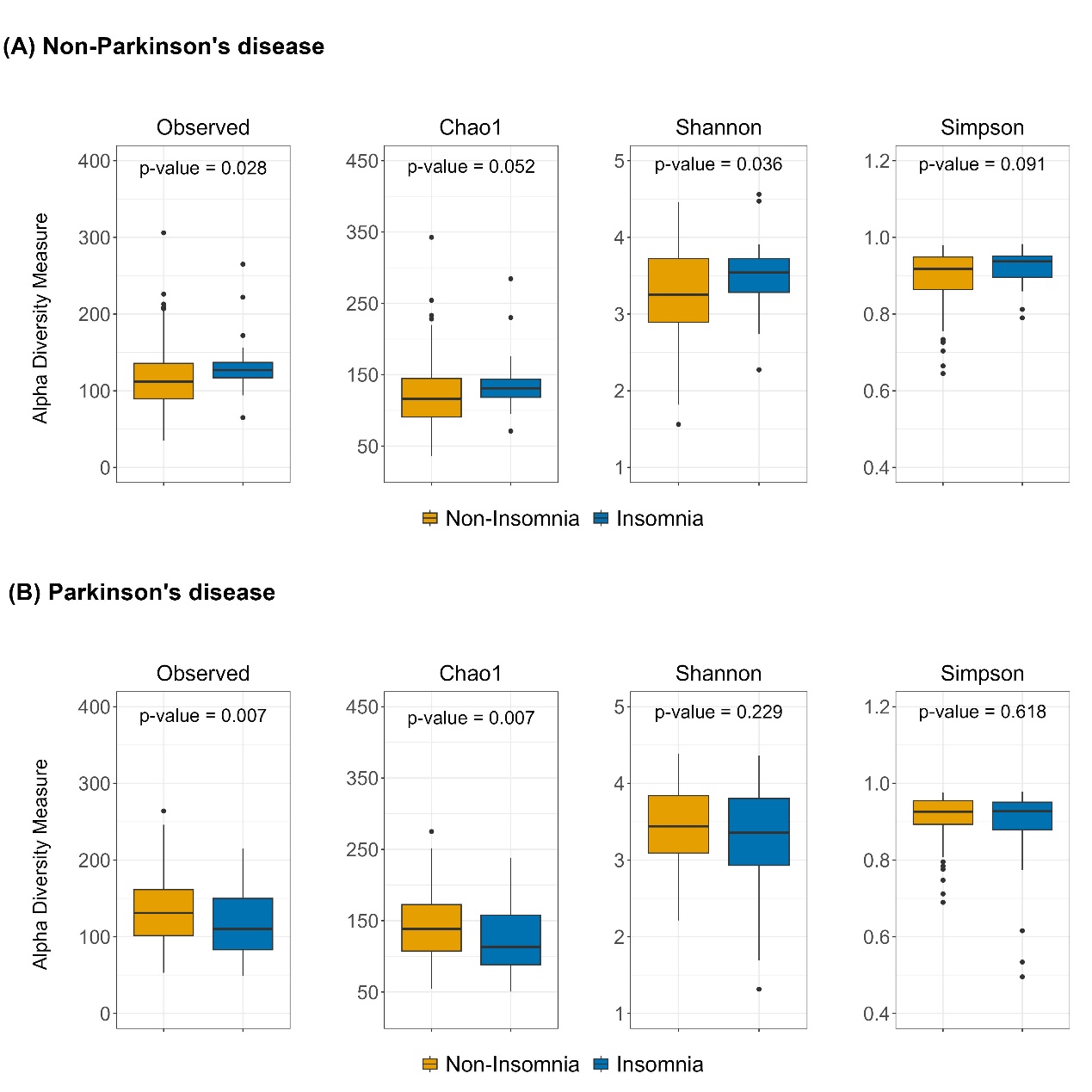


**Figure S1. Alpha diversity differences adjusted sequence depth between insomnia and non-insomnia groups in both (A) non-PD and (B) PD groups.** The boxplots show the alpha diversity of the bacterial communities by means of observed amplicon sequence variants (ASVs), and Chao1, Shannon, and Simpson indexes. Median, and also lower and upper quartiles are shown on the plots.

**Table S2. Relationship between Alpha Diversity Indices and Sequencing Depth in (a) non-PD and (b) PD group.** P-values in the first row were obtained through analysis of variance. Rho represents the Spearman correlation coefficient between sequencing depth and diversity indices, with the corresponding P-values shown in the last row.

**(a) non-PD group**

|  | Observed | Chao1 | Shannon | Simpson |
| --- | --- | --- | --- | --- |
| P-value | <0.001 | <0.001 | 0.052 | 0.498 |
| Rho | 0.380 | 0.429 | 0.082 | 0.041 |
| P-value of rho | <0.001 | <0.001 | 0.363 | 0.646 |

**(b) PD group**

|  | Observed | Chao1 | Shannon | Simpson |
| --- | --- | --- | --- | --- |
| P-value | <0.001 | <0.001 | 0.001 | 0.076 |
| Rho | 0.422 | 0.465 | 0.249 | 0.198 |
| P-value of rho | <0.001 | <0.001 | <0.001 | 0.007 |


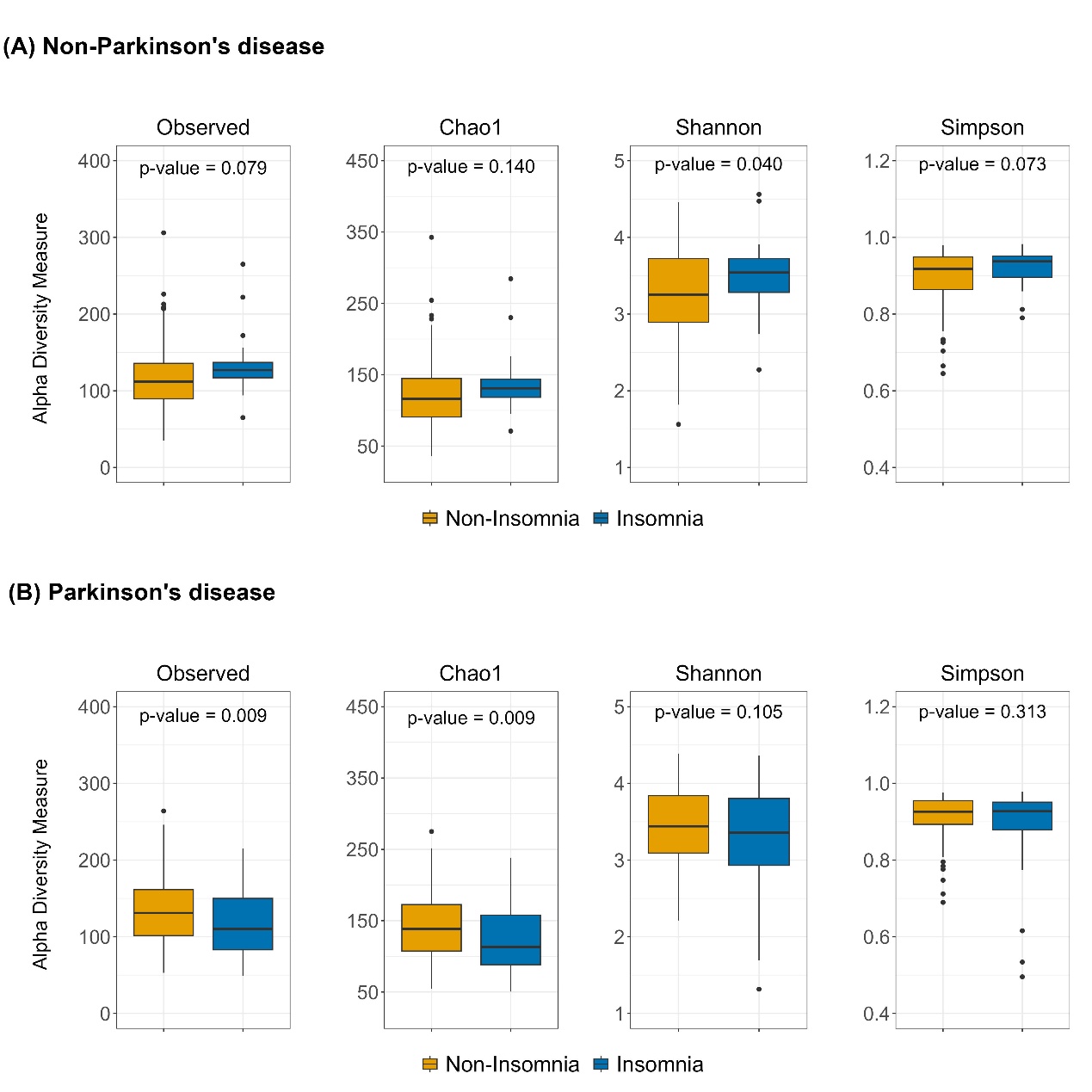


**Figure S2.** **Alpha diversity differences adjusted depression between insomnia and non-insomnia groups in both (A) non-PD and (B) PD groups.** The boxplots show the alpha diversity of the bacterial communities by means of observed amplicon sequence variants (ASVs), and Chao1, Shannon, and Simpson indexes. Median, and also lower and upper quartiles are shown on the plots.


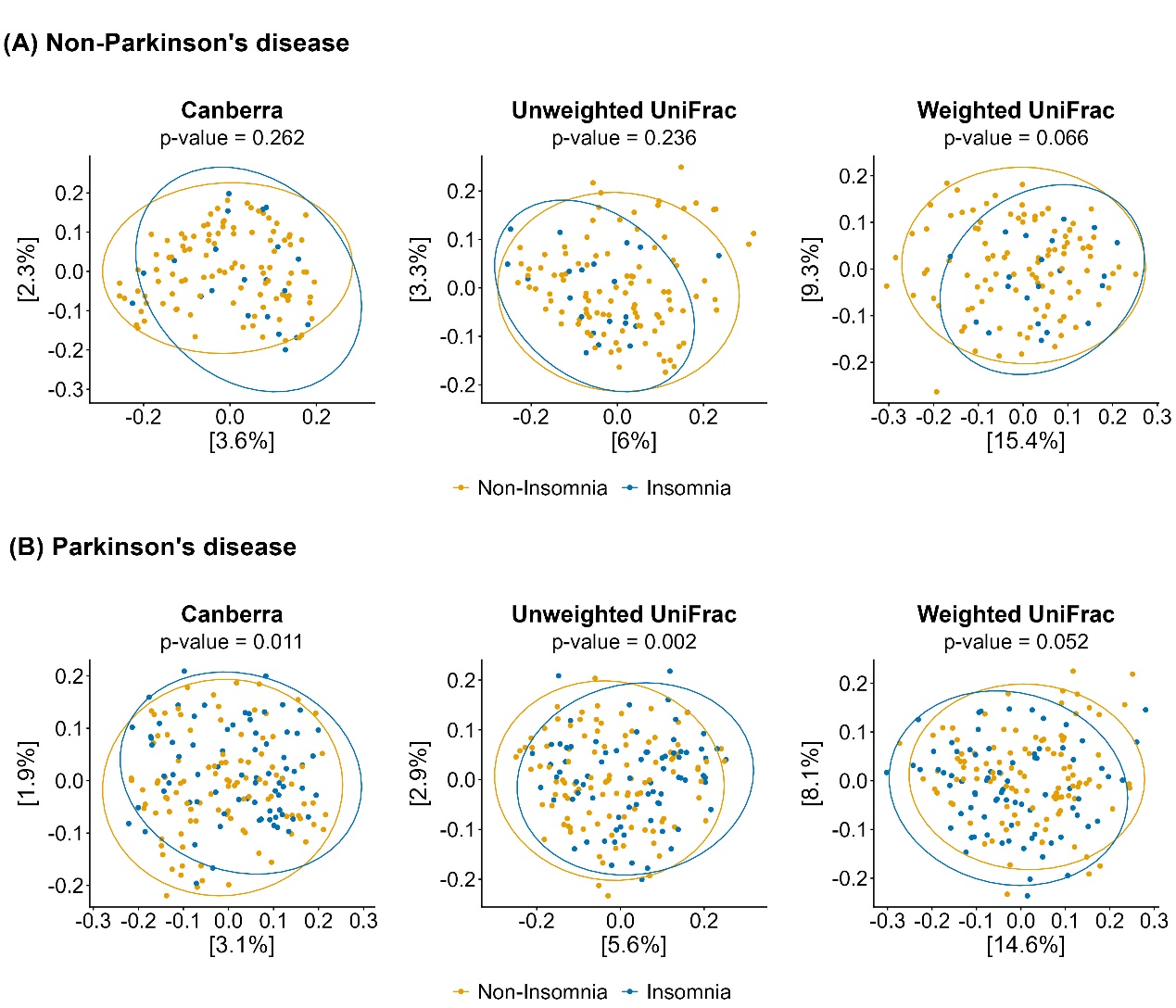


**Figure S3. Beta diversity differences adjusted depression between insomnia and non-insomnia groups in both (A) non-PD and (B) PD groups.** The PCoA plots show the following 3 distance measures: Canberra, unweighted unique fraction metric (UniFrac), and weighted UniFrac.


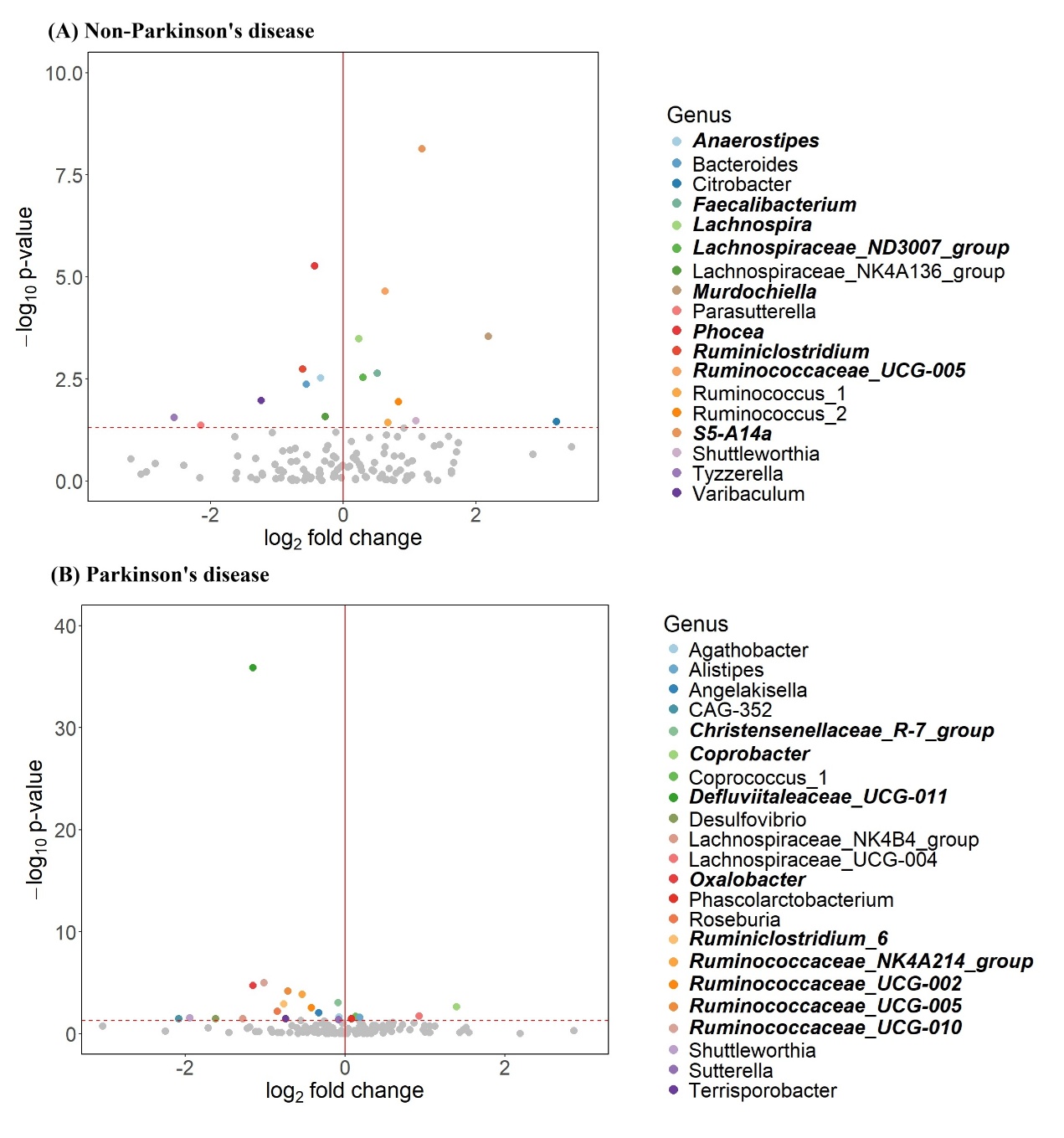


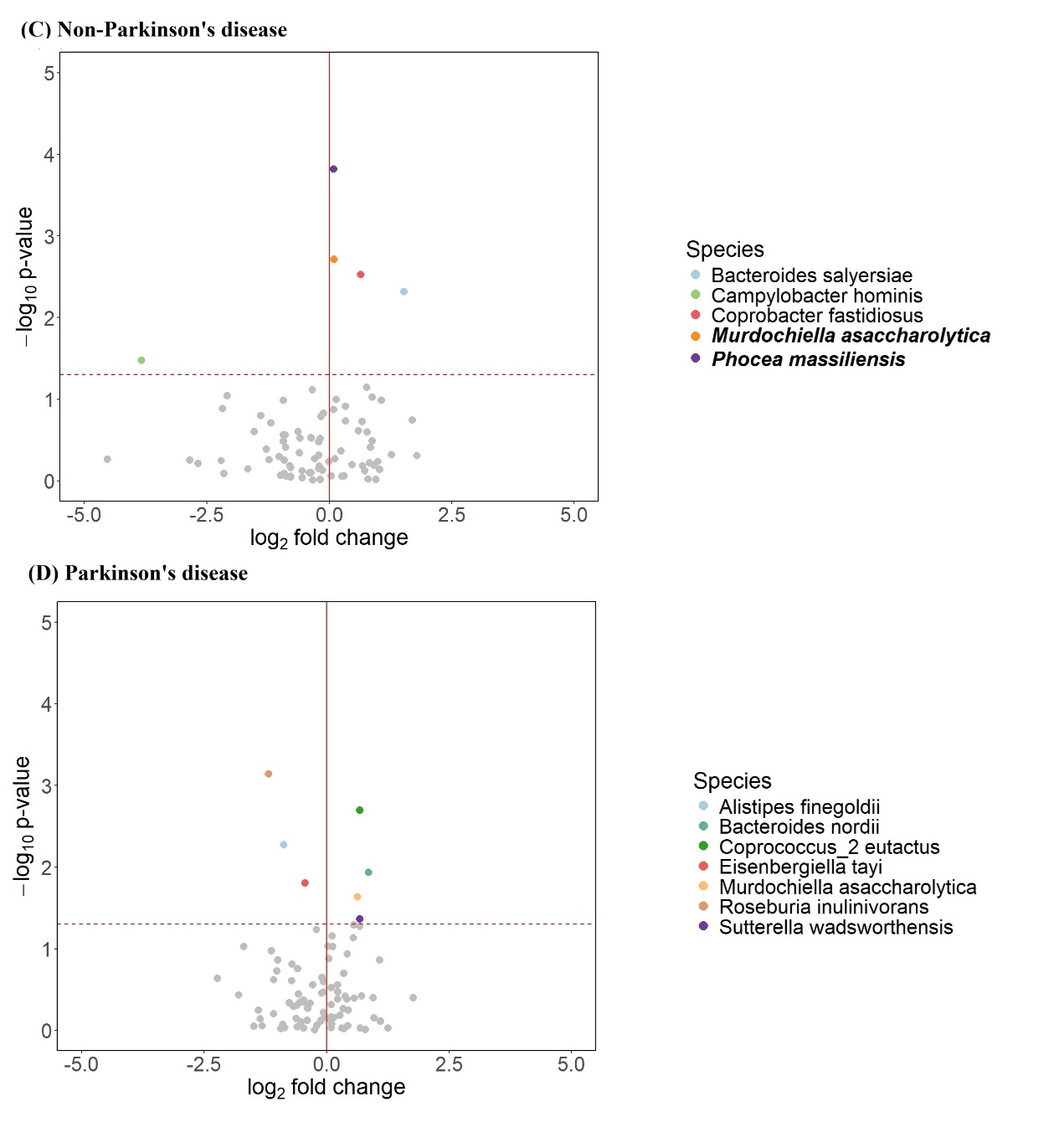


**Figure S4. Differentially abundant taxa adjusted depression before FDR correction.** Volcano plots show microbial taxa associated with insomnia in (A) non-PD and (B) PD groups at the genus level, and (C) non-PD and (D) PD groups at the species level. Each point represents a taxon, with log_2_ fold change on the x-axis and -log_10_ p-value on the y-axis. The red vertical line indicates no change, while the horizontal dashed line marks the significance threshold. Colored points denote significant taxa before FDR correction, and bolded names indicate significance after FDR correction.


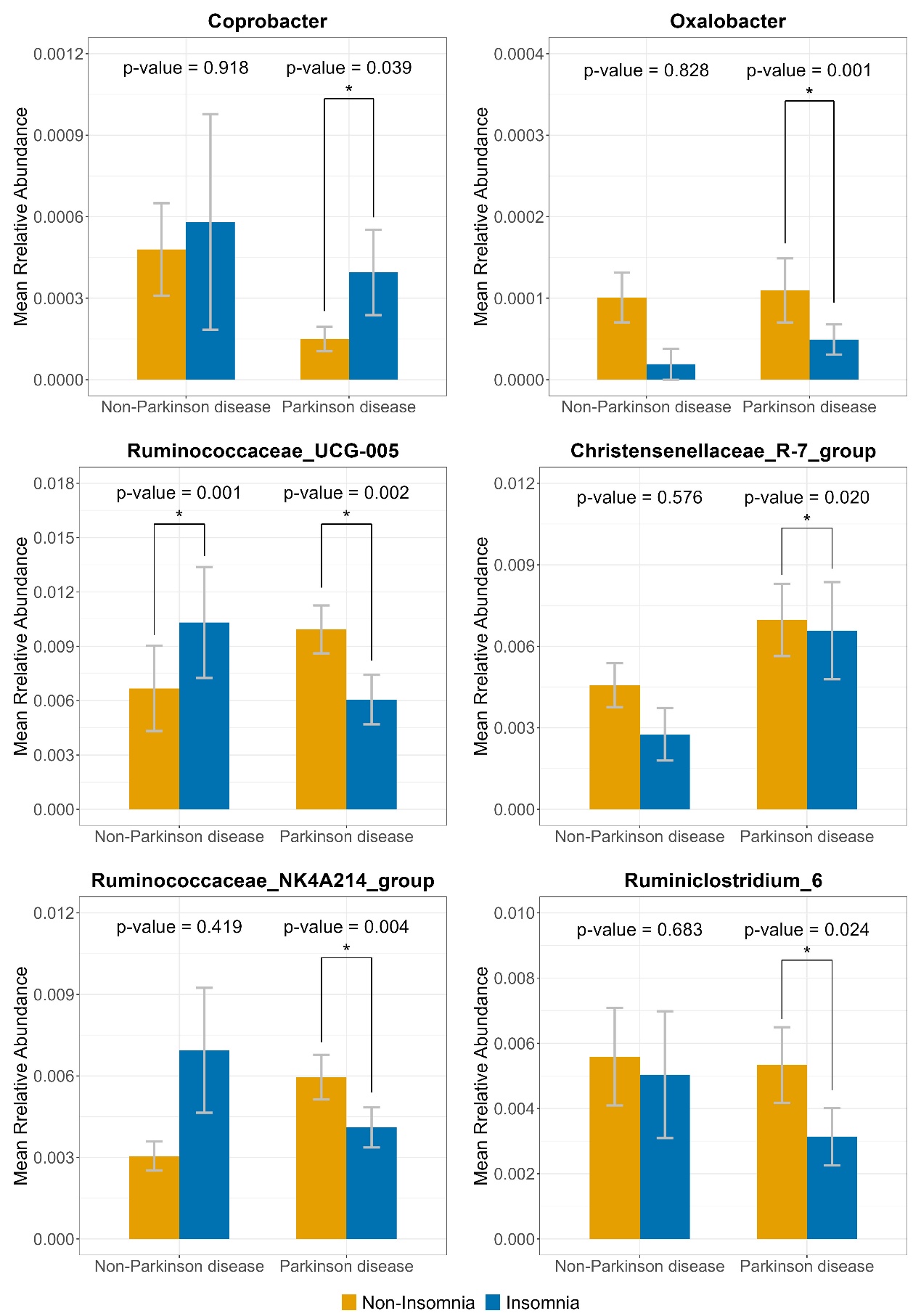


**Figure S5. Relative abundance of insomnia-associated genera adjusted depression in PD and non-PD groups.** Mean relative abundance of six insomnia-associated genera in both PD and non-PD groups, comparing insomnia and non-insomnia participants. Significant differences were observed in the PD group, but these genera were either non-significant or showed opposite trends in the control group. Error bars represent mean ± standard deviation.


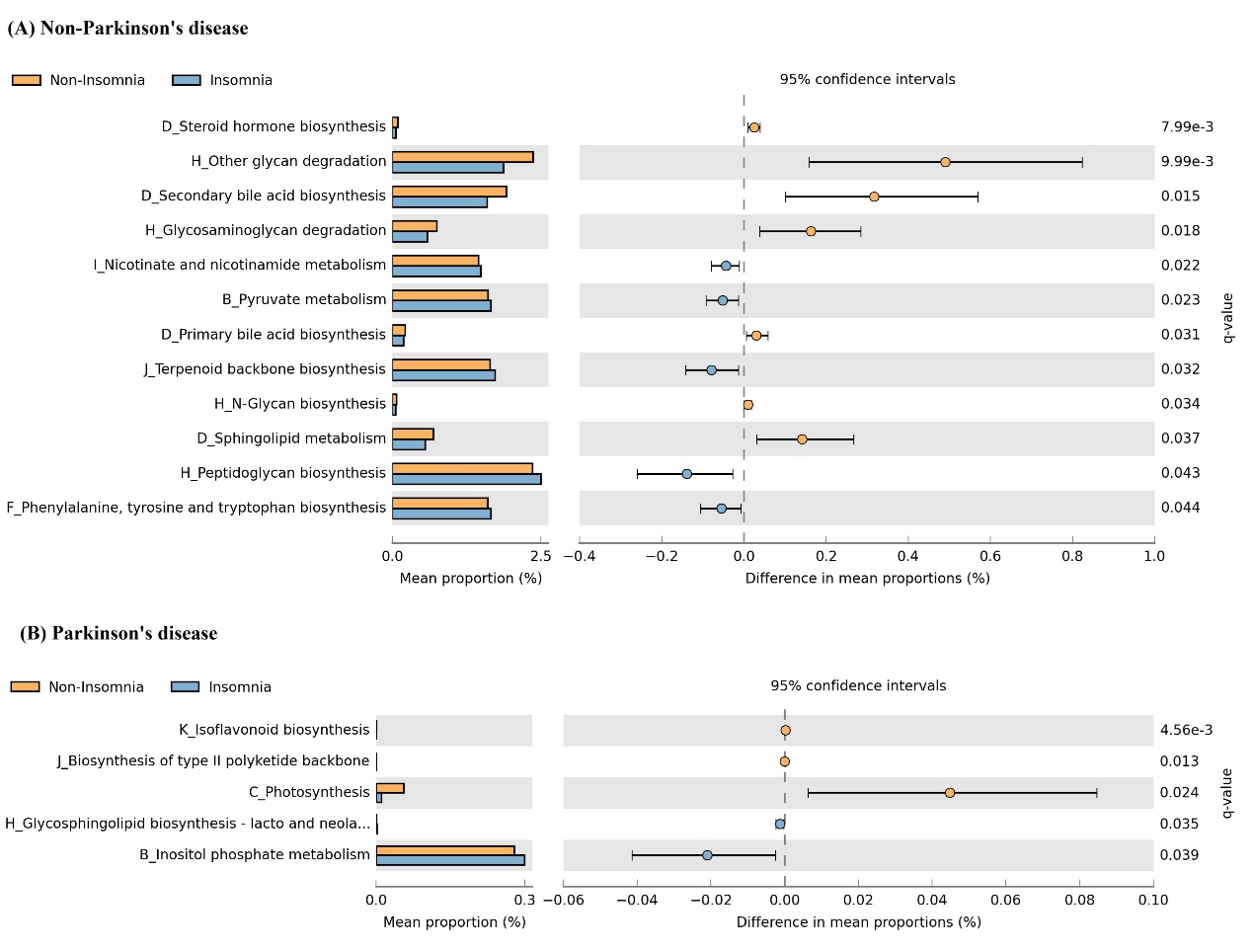


**Figure S6. Functional pathway enrichment adjusted depression in insomnia participants.** Functional pathways significantly enriched in insomnia participants differed between PD and non-PD groups. The control group showed enrichment in twelve pathways related to metabolism and biosynthesis, while the PD group exhibited enrichment in only five pathways linked to neuroinflammation and cell signaling. No overlapping pathways were observed between groups.

| **Table S3.** Comparing complete subjects and excluded subjects on demographic variables. | | | | |  |
| --- | --- | --- | --- | --- | --- |
|  | **Complete (N = 310)** | **Excluded (N = 20)** | **p-value** | |  |
| **PD** |  |  | 0.497 | |  |
| No | 125 (40.3%) | 6 (30.0%) |  |  |  |
| Yes | 185 (59.7%) | 14 (70.0%) |  |  |  |
| **Gender** |  |  | 0.741 | |  |
| Female | 135 (43.5%) | 10 (50.0%) |  |  |  |
| Male | 175 (56.5%) | 10 (50.0%) |  |  |  |
| **Age ＞ 65** | |  |  | 0.047 | |
| No | 106 (34.2%) | 2 (10.0%) |  |  |  |
| Yes | 204 (65.8%) | 18 (90.0%) |  |  |  |
